# Supplementary material for: Influence of the Bile Acid Transporter Genes ABCB4, ABCB8, and ABCB11 and the Farnesoid X Receptor on the Response to Ursodeoxycholic Acid in Patients with Nonalcoholic Steatohepatitis
Source: J Pers Med. 2023 Jul 24;13(7):1180. doi: 10.3390/jpm13071180 (PMC10381823; doi:10.3390/jpm13071180)
Supplement: Supplementary file 1 [file jpm-13-01180-s001.zip › jpm-2482886-supplementary.pdf]

Journal of Personalized Medicine

Tables:

Figures:

Supplements:

Polymorphism per patient	Absolute decrease ALT Median (U/l) (min-max)	p-value	Relative decrease ALT Median % (min-max)	p-value
1 (n=1)	32 (32–32)	0.205	110 (110-110)	0.262
2 (n=3)	-9 (-45–25.8)		-6.87 (-64.3–159)	
3 (n=6)	-28 (-44.4–-7)		-38.5 (-68.8–-12.3)	
4 (n=14)	-10.3 (-60–9)		-26.1 (-52.5–36)	
5 (n=4)	-0.5 (-60–19.2)		2.59 (-71.4–24.6)	

Polymorphism per patient	Absolute decrease GGT Median (U/l) (min-max)	p-value	Relative decrease GGT Median % (min-max)	p-value
1 (n=1)	20 (20–20)	0.331	22 (22–22)	0.812
2 (n=3)	-57.6 (-137–173)		-69.2 (-72.2–59)	
3 (n=6)	-107 (-360–-14)		-64.5 (-80.9–-12.7)	
4 (n=14)	-72.8 (-1084–96)		-54.6 (-84–61.8)	
5 (n=4)	-284 (-1105–-68.4)		-35.7 (-94.4–-22.5)	

Polymorphism per patient	Absolute decrease Fib 4 Median (min-max)	p-value	Relative decrease Fib 4 Median % (min-max)	p-value
1 (n=1)	3.26 (3.26–3.26)	0.131	182 (182–182)	0.203
2 (n=3)	0.069 (-0.882–0.151)		11.9 (-28.4–23.3)	
3 (n=6)	-0.012 (-0.209–0.114)		-2.14 (-27.2–13)	
4 (n=14)	0.162 (-0.81–5.85)		16.3 (-62.4–∞)	

5 (n=4)	0.416 (-0.087–2.14)		33.1 (-18.1–50.9)	

Table S1: Absolute and relative decrease of ALT, GGT, and Fib-4 per number of polymorphism per patients. P-values have been calculated with Kruskal-Wallis Test. Positive values show an increase and negative values a decrease.

	1-2 polymorphism (n=4)<br><br>Median (min-max)	3-5 polymorphism (n=24)<br><br>Median	p-value (Wilcoxon)
ALT (U/l)	8.4 (-45–32)	-11.8 (-60–19.2)	0.29
GGT (U/l)	-18.8 (-137–173)	-96.5 (-1105–96)	0.14
Fib-4	0.11 (-0.882–3.26)	0.112 (-0.81–5.85)	1

Table S2: Absolute decrease of ALT and GGT in patients with 1-2 concurrent polymorphisms compared to patients with 3-5 polymorphisms. P-values were calculated using paired Wilcoxon tets. Positive values indicate an increase and negative values a decrease.

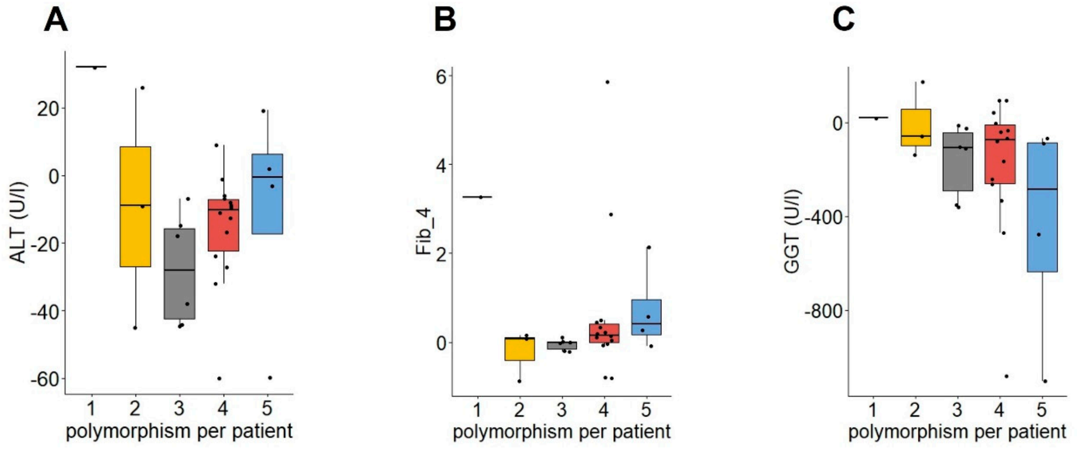

Figure S1: ALT (A) and Fib-4 (B) show the highest decrease in patients with 3 concurrent polymorphisms. while GGT (C) decreased with every polymorphism without reaching significance. Due to the small number of patients per group pairwise comparisons were not calculated.
